# Effects of Qi-Fang-Xi-Bi-Granules on Cartilage Morphology and C/ebp*α* Promoter Methylation in Rats with Knee Osteoarthritis

**DOI:** 10.1155/2018/2074976

**Published:** 2018-01-28

**Authors:** Xinxin Wang, Peng Shen, Dezhi Tang, Hao Xu, Hongfu Qiu, Tao Wu, Xiang Gao

**Affiliations:** ^1^Department of Surgery, Huadong Hospital Affiliated to Fudan University, Shanghai 200040, China; ^2^Spine Research Institute, Shanghai University of Traditional Chinese Medicine, Shanghai 200032, China

## Abstract

**Objective:**

To investigate the effects of Qi-Fang-Xi-Bi-Granules (QFXBGs) on cartilage morphology and methylation of C/ebp*α* (CCAAT/enhancer binding protein*α*) at the promoter region.

**Methods:**

Knee osteoarthritis (KOA) modeling was performed in rats in accordance with Hulth's method, and control group received sham operation. Eight weeks after KOA modeling, the rats in the KOA modeling group were further divided into 6 groups. Each group was given the appropriate drug. After 8 weeks, half of the rats were used for Micro-CT scan, HE staining, ABH/OG staining, immunohistochemistry, and TUNNEL staining of the knee joint tissue, and the other half were used to examine C/ebp*α* promoter methylation.

**Results:**

The three dose groups of QFXBGs all showed lower degrees of surface fissures and flaking, thicker cartilage layer, and restored chondrocyte and subchondral bone morphology, compared with the KOA model group. C/ebp*α*-22 promoter methylation levels in the high- and low-dose groups were significantly higher than that in the KOA modeling group (*p* < 0.05), while C/ebp*α*-2 promoter methylation level in the medium-dose group was significantly higher than that in the KOA modeling group (*p* < 0.05).

**Conclusions:**

QFXBGs may alleviate articular cartilage degeneration through promoting C/ebp*α*-2 or C/ebp*α*-22 methylation at specific promoter sites.

## 1. Introduction

Knee osteoarthritis (KOA) is a degenerative knee disease that commonly occurs in the ageing population. It is usually derived from pathology of the cartilage, subchondral bone and synovial membrane of the knee joint. KOA is clinically characterized by progressive knee pain, swelling, stiffness, restricted joint range of motion, and even joint deformity under serious conditions, resulting in loss-of-function of the joint, largely affecting the life quality of patients and posing a great economic burden [1]. Currently, there is no treatment that can completely cure KOA. All therapies are aimed at ameliorating symptoms and improving joint functions. KOA is a multifactorial disease. Apart from living style and healthy conditions, it is also subjected to other factors like genetic alterations and environmental conditions.

Changes in genetic modulation in cartilage may be implicated in the pathogenesis and progression of KOA. DNA methylation is one such player that has receiving increasing attention. DNA methylation is a typical form of epigenetic modification. It involves the formation of 5-methylcytosine through transference of a methyl group from S-adenosyl methionine to the 5th atom of the cytosine ring, a process catalyzed by DNA methyltransferase. DNA methylation plays critical roles in embryo development, X chromosome inactivation, regulation of gene expression, gene imprinting and silencing, and tumorigenesis [2].

In this study, we investigated the alterations of C/ebp*α* (CCAAT/enhancer binding protein *α*) promoter methylation in the presence of Qi-Fang-Xi-Bi-Granules (QFXBGs), to elucidate the mechanisms underlying the effect of QFXBG on cartilage.

C/ebp*α* is the first C/ebp family member found in mouse collagen promoter in the late 1980s. C/ebp*α* is a leucine-zipper transcriptional factor with a highly conserved C-terminal.* C/ebpα* binding of DNA requires dimerization of this protein [3]. C/ebp*α* is present in multiple types of cell and plays an important role in cell proliferation and differentiation and ontogenesis, as well as tumorigenesis and tumor development.

Chinese medicinal herbs had been used in the treatment for osteoarthritis for a long time with few side effects. Qi-Fang-Xi-Bi prescription is a composition of Chinese herbal medicines that has shown promising efficacy in alleviating knee joint diseases in the past two decades of clinical practice in Huadong Hospital Affiliated to Fudan University and Longhua Hospital Affiliated to Shanghai University of Traditional Chinese Medicine [4, 5]. However, although QFXBGs have entered standardized manufacture and are commercially available at present [6], their effects on articular cartilage and subchondral bone under the condition of KOA have not been reported. In this study, we investigated the morphological alterations of articular cartilage and subchondral bone in the presence of QFXBGs as well as the effects of QFXBGs on C/ebp*α* promoter methylation in rats with KOA, by using approaches of Micro-CT, HE staining, ABH/OG staining, immunohistochemistry, TUNNEL staining, and methylation sequencing. This work was set out to elucidate the role of QFXBGs in alleviating knee degeneration and provide basis for clinical treatment of KOA.

## 2. Materials and Methods

### 2.1. KOA Modeling in Rats

Seventy SPF-grade Sprague-Dawley rats of 8 weeks (Beijing Vital River Laboratory Animal Technology Co., Beijing, China) were randomly assigned to sham group (*n* = 10) and KOA modeling group (*n* = 70) and cage-raised at 21-22°C under a 12 h : 12 h light/dark cycle with free access to food and sterile tap water. After one-week acclimation, KOA modeling was performed in accordance with Hulth's method after 12 h fasting. The control group only received a 1 cm longitudinal incision on the skin of the medial right-posterior knee. The incisions were sutured and chlortetracycline ointment was applied onto and around the suture to prevent infection. Then the rats were allowed free activity and feeding. Further agent administration was made after 8 weeks [7]. All the experimental procedures were approved by the Animal Use and Care Committee of Shanghai University of Chinese Traditional Medicine.

### 2.2. Rat Subgrouping and Administration

Three rats died within 8 weeks after modeling, leaving 8 rats in the control group and 59 rats in the modeling group. Rats in the modeling group were further divided into 6 groups: negative control group (KOA model group, *n* = 9), Celebrex treatment group (0.013 g/kg/day, Celecoxib capsules®, Cat. number L61633, Pfizer, Dalian, China) (*n* = 9), Viartril-S treatment group (0.100 g/kg/day, Sulfated Glucose Capsules®, Cat. number 1411244; Rottapharm Ltd., Ireland) (*n* = 10), QFXBG low-dose group (0.836 g/kg/day) (*n* = 10), QFXBG medium-dose group (1.672 g/kg/day) (*n* = 10), and QFXBG high-dose group (3.344 g/kg/day) (*n* = 10). All the agents were dissolved in normal saline. Rats in the control and negative groups received intragastric administration of the same volume of normal saline. All the agents were administered for consecutive 8 weeks, after which they were sacrificed.

Qi-Fang-Xi-Bi-Granules are composed of Astragali radix (Huangqi) 15 g, Stephaniatetrandrae radix (Fangji) 12 g, Achyranthisbidentatae radix (Niuxi) 12 g, Angelicaesinensis radix (Danggui) 9 g, Atracylodisrhizoma (Cangzhu) 12 g, Eupolyphagaesinensis walker whole body (Dibiechong) 12 g, and Epimediiherba (Xianlingpi) 12 g.

### 2.3. Tissue Preparation

Five rats in each group were randomly selected for cartilage collection. Joint cavity of the right-posterior knee was opened under anesthesia. The articular cartilage was gently cut by a surgical knife, during which caution was taken to avoid cutting the subchondral bone. The cartilage collected was stored in formalin for detection of C/ebp*α* promoter methylation in MethylTarget. The remaining rats in each group were used to collect knee joint tissue from the right hind limb after removal of the surrounding soft tissue including muscles and fat, which spanned ±2 cm to the knee joint. The knee joint tissue was fixed in formalin for 48 h and then stored in 75% ethanol until use for Micro-CT scan, HE staining, ABH/OG staining, immunohistochemistry, and TUNNEL staining [7].

### 2.4. Sample Processing

After Micro-CT (Scanco, model vivaCT80), the knee joint tissue was decalcified in 14% EDTA (PH 7.40 ± 0.05) for 4 weeks, with replacement of the decalcification solution every 2-3 days. After completion of decalcification, dehydration, diffuse wax, and embedding were performed, and 5 *μ*m sections were cut for HE staining, ABH/OG staining, immunohistochemistry, and TUNNEL staining [7].

The cartilage was used for detection of methylation in the CpG island of C/ebp*α* promotor based on the second-generation high-throughput sequencing platform, in combination with bisulfite treatment and generation of DNA methylation mapping by bioinformatics analysis [8, 9].

### 2.5. Bisulfite Conversion and Multiplex Amplification

DNA methylation level was analysis by MethylTarget™, an NGS-based multiple targeted CpG methylation analysis method. Specifically, the genomic regions of interest were analyzed and transformed to bisulfite-converted sequences by geneCpG software. PCR primer (Table 1) sets were designed with the Methylation Primer software from bisulfate converted DNA [7].

Genomic DNA (400 ng) was subjected to sodium bisulfite treatment using EZ DNA Methylation™-GOLD Kit (Zymo Research) according to manufacturer's protocols. Multiplex PCR was performed with optimized primer sets combination. A 20 *μ*l PCR reaction mixture was prepared for each reaction and included 1x reaction buffer (Takara), 3 mM Mg2+, 0.2 mMdNTP, 0.1 *μ*M of each primer, 1 U HotStarTaqpolymerase (Takara), and 2 *μ*l templates DNA. The cycling program was 95°C for 2 min; 11 cycles of 94°C for 20 s, 63°C for 40 s with a decreasing temperature step of 0.5°C per cycle, and 72°C for 1 min and then followed by 24 cycles of 94°C for 20 s, 65°C for 30 s, and 72°C for 1 min; 72°C for 2 min [7].

### 2.6. Index PCR

PCR reactions were diluted and amplified using indexed primers. Specifically, a 20 *μ*l mixture was prepared for each reaction and included 1x reaction buffer (NEB Q5TM), 0.3 mMdNTP, 0.3 *μ*M of F primer, 0.3 *μ*M of index primer, 1 U Q5TM DNA polymerase (NEB), and 1 *μ*L diluted template. The cycling program was 98°C for 30 s; 11 cycles of 98°C for 10 s, 65°C for 30 s, and 72°C for 30 s; 72°C for 5 min. PCR reactions (170 bp–270 bp) were separated by agarose electrophoresis and purified using QIAquick Gel Extraction kit (QIAGEN) [7].

### 2.7. Sequencing

Libraries from different samples were quantified and pooled together, followed by sequencing on the IlluminaMiSeq platform according to manufacturer's protocols. Sequencing was performed with a 2 × 300 bp paired-end mode [7].

### 2.8. Statistics

Quality control of sequencing reads was performed by FastQC. Filtered reads were mapped to genome by blast after reads recalibration with USEARCH. Data of methylation rate are presented as mean ± SD and were analyzed with independent *t*-test or *U* test using Perl programming language; *p* < 0.05 was considered as statistically significant. Graphs were made with the GraphPad Prism 5.0 software [7].

## 3. Results

Of the 70 SD rats employed, three died after KOA modeling, including 2 in the sham operation group and 1 in the modeling group. Therefore finally 67 rats were finally used for subsequent experiments and data analysis.

### 3.1. Micro-CT, HE Staining, and ABH/OG Staining Results

**Figure 1:**
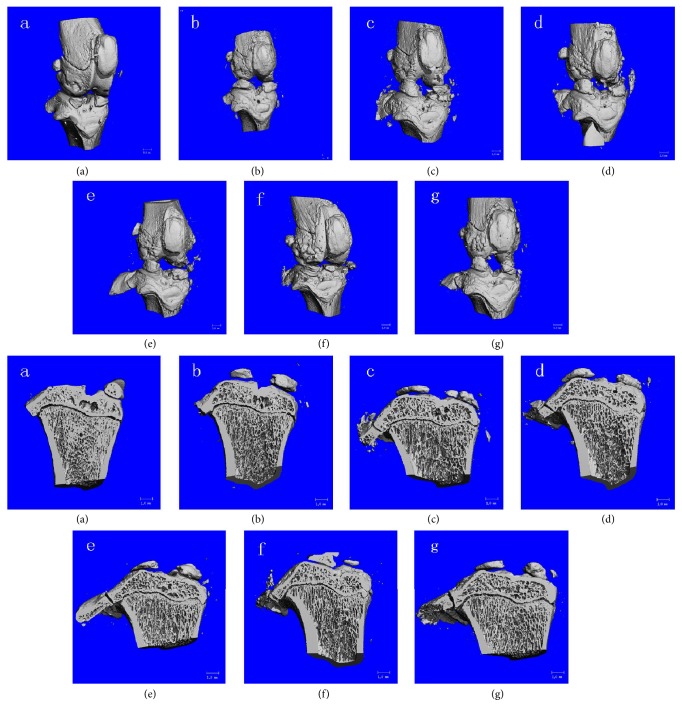
Micro-CT on knee joint tissue. (a) Normal control group; (b) KOA model group; (c) Celebrex group; (d) Viartril-S group; ((e)–(g)) low-dose (e), medium-dose (f), and high-dose (g) QFXBG groups.

Results of knee joint Micro-CT (Figure 1) showed a smooth cartilage surface of the knee joint (Figure 1(a)), with no apparent formation of osteophyte. As seen in the coronal section (Figure 1(b)), the cancellous bone trabeculae in the subchondral bone were thick and uniformly arrayed. In the KOA model group, the cartilage had a rough surface, with focal surface fissures and flaking, exposure of the subchondral bone, and formation of round or oval osteophytes along the lining of joint (Figure 1(a)). The coronal section (Figure 1(b)) showed thinning of the cartilage layer, small and disorganized trabeculae, and cystic degeneration of the subchondral bone in KOA.

**Figure 2:**
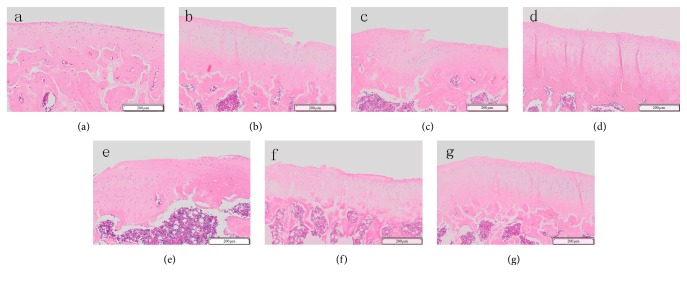
HE staining of knee joint tissue. (a) Normal control group; (b) KOA model group; (c) Celebrex group; (d) Viartril-S group; ((e)–(g)) low-dose (e), medium-dose (f), and high-dose (g) QFXBG groups.

**Figure 3:**
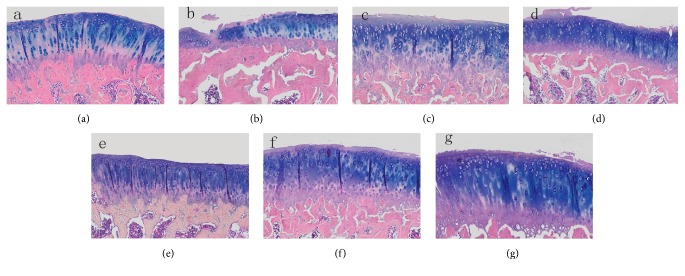
ABH/OG staining of knee joint tissue. (a) Normal control group; (b) KOA model group; (c) Celebrex group; (d) Viartril-S group; ((e)–(g)) low-dose (e), medium-dose (f), and high-dose (g) QFXBG groups.

In the sham operation group, HE staining (Figure 2(a)) also showed a smooth cartilage surface, evenly-light-pink-colored chondrocyte matrix, and blue nuclei. The cartilage layers and structure were clearly seen. The superficial layer was composed of flat chondrocyte and the middle layer was composed of uniformly arrayed round chondrocytes, while the deep layer and the calcified layer contained hyperplastic chondrocytes. ABH/OG staining (Figure 3(a)) revealed that the chondrocyte matrix was evenly stained in blue. In the KOA model group, HE staining (Figure 2(b)) showed thinning of the cartilage layer with significant surface fibrosis, chondrocyte necrosis, unclear nucleus and cytoplasm, a rough cartilage surface with fissures and flaking (even cartilage loss), exposure of the subchondral bone, uneven coloration, unclear structure and tidal line of the cartilage layer, proliferation of chondrocytes, disorganized cell alignment, presence of large amount of cell clusters, and blood vessel passing through the tide line. ABH/OG staining (Figure 3(b)) revealed weak staining and many regions with negative staining, and strong fibrosis. These pathological alterations indicate the successful establishment of KOA model in rats with Hulth's method. When compared to the KOA control group, all the drug treatment groups showed alleviated KOA pathology.

As seen from the Micro-CT image (Figure 1), the high-dose QFXBG group had a smoother cartilage surface and less osteophytes (Figure 1(g)), as well as having a thicker cartilage layer and better conditions in size, density, and arrangement of cancellous bone trabeculae in the subchondral bone (Figure 1(g)), than the other two dose groups. The overall pathology of cartilage and subchondral bone was comparable between the medium-dose and low-dose QFXBG groups. All the three doses of QFXBGs showed obvious alleviation of KOA pathology compared to the KOA control group.

Micro-CT revealed that both the Celebrex treatment group (Figure 1(c)) and the Viartril-S group (Figure 1(d)) had ameliorated knee joint injury than the KOA model group. In particular, however, Viartril-S performed worse than high-dose QFXBG, comparably to the medium-dose QFXBG, and better than low-dose QFXBG, in restoring the thickness of cartilage layer, the size and arrangement of cancellous bone trabeculae, and proliferation of osteophytes. The Celebrex performed worse than high- and medium-dose QFXBGs but was comparable to low-dose QFXBG.

In sum, the results of HE staining (Figure 2) and ABH/OG histological staining (Figure 3) showed a less severity of KOA in QFXBG high-dose group than in the medium- and low-dose groups. The effects of high-dose QFXBG were comparable to Viartril-S and better than Celebrex.

### 3.2. Immunohistochemical and TUNNEL Staining Results

**Figure 4:**
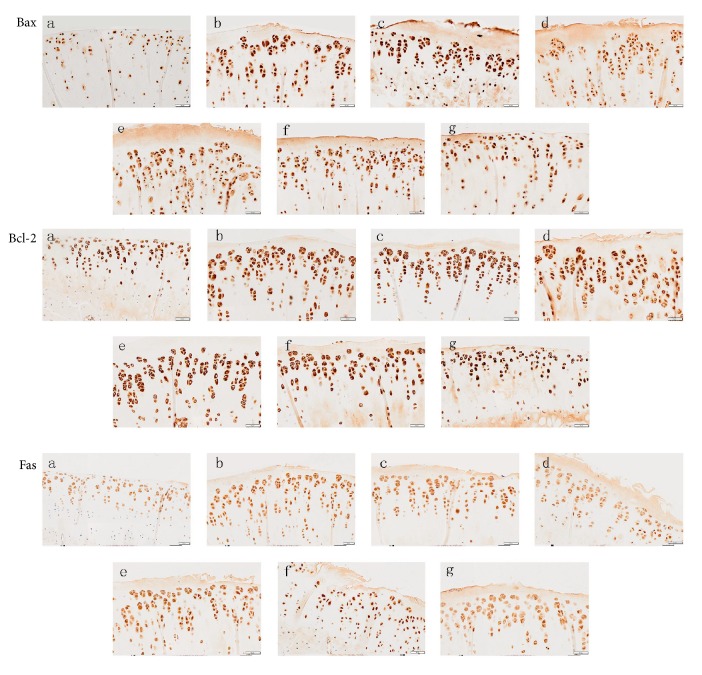
Immunohistochemistry of knee joint tissue. (a) Normal control group; (b) KOA model group; (c) Celebrex group; (d) Viartril-S group; ((e)–(g)) low-dose (e), medium-dose (f), and high-dose (g) QFXBG groups.

**Figure 5:**
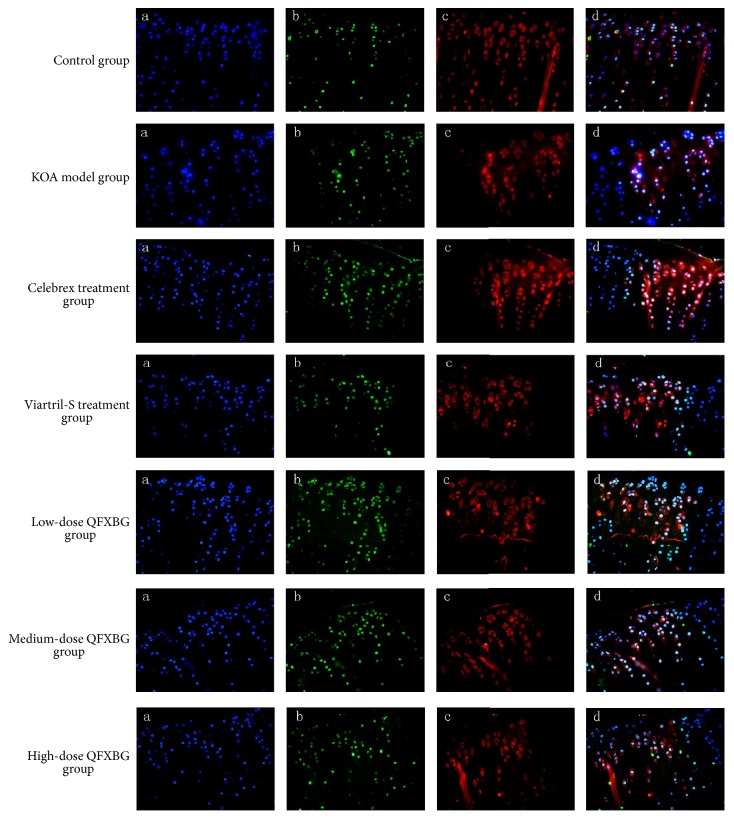
TUNNEL staining of knee joint tissue. (a) shows chondrocyte nucleus; (b) shows apoptotic nucleus; (c) shows cytoplasm of chondrocytes; (d) is a merge of (a), (b), and (c).

Results of immunohistochemical staining showed significantly increased Bax, Bcl-2, and Fas in the KOA control group than in the sham operation group (Figure 4). When compared with the KOA control group, these inflammatory factors were all significantly decreased in the Celebrex treatment group, the Viartril-S group, and the QFXBG high-dose group (Table 2), while the medium and low doses of QFXBG did not substantially decrease these factors. TUNNEL staining (Figure 5, Table 2) revealed significantly increased apoptosis of chondrocytes in the KOA control group than the sham operation group. When compared with the KOA control group, the high-dose QFXBG significantly inhibited apoptosis, while Celebrex, Viartril-S, and the medium and low doses of QFXBG cannot inhibit apoptosis.

### 3.3. C/ebp*α* Promoter Methylation in the CpG Island

**Figure 6:**
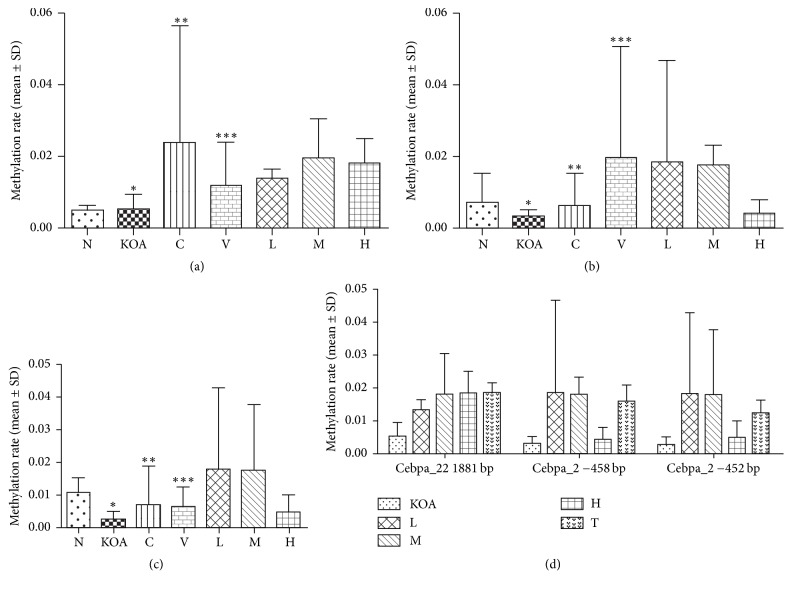
C/ebp*α* methylation at various sites. N stands for normal control group, KOA stands for KOA model group, C stands for Celebrex group, V stands For Viartril-S group, L stands for low-dose QFXBG group, M stands for medium-dose QFXBG group, H stands for high-dose QFXBG group, and T stands for the combination of L M and H Groups; (a) C/ebp*α*-22 with the CpG islands 1881 bp to the initiation codon; (b) C/ebp*α*-2 with the CpG islands −458 bp to the initiation codon; (c) C/ebp*α*-2 with the CpG islands −452 bp to the initiation codon; (d) differences in methylation rates among groups QFXBGs and KOA model group. ^*∗*^*p* = 0.4907, ^*∗*^*p* = 0.3400, and ^*∗*^*p* = 0.0054 versus N group; ^*∗∗*^*p* > 0.05 and ^*∗∗∗*^*p* > 0.05 versus KOA group.

When compared with the sham operation group, the KOA control group had higher ratios of C/ebp*α*-2 methylation at 4 bp (*p* > 0.05) and 10 bp (*p* = 0.0054) from the start codon, as well as methylation ratio of C/ebp-22 at 1419 bp from the start codon (*p* > 0.05) (Figure 6). Celebrex, Viartril-S, and QFXBG treatment (pooled effect) all improved these ratios, but statistical significance was achieved only for QFXBG treatment (*p* = 0.0365, *p* = 0.0495, and *p* = 0.0115). More specifically, high and low doses of QFXBG significantly improved C/ebp*α*-22 methylation at 1419 bp (*p*_low_ = 0.0088, *p*_high_ = 0.0094) (Figure 6(d), Table 3), while the medium-dose QFXBG significantly increased C/ebp*α*-2 methylation at 4 bp (*p*_Z_ = 0.0005). There were no significant differences in C/ebp*α*-2 and C/ebp*α*-22 methylation among the low, medium, and high doses of QFXBGs (Figure 6(d)).

### 3.4. The *β*-Catenin Immunohistochemical Staining Results

**Figure 7:**
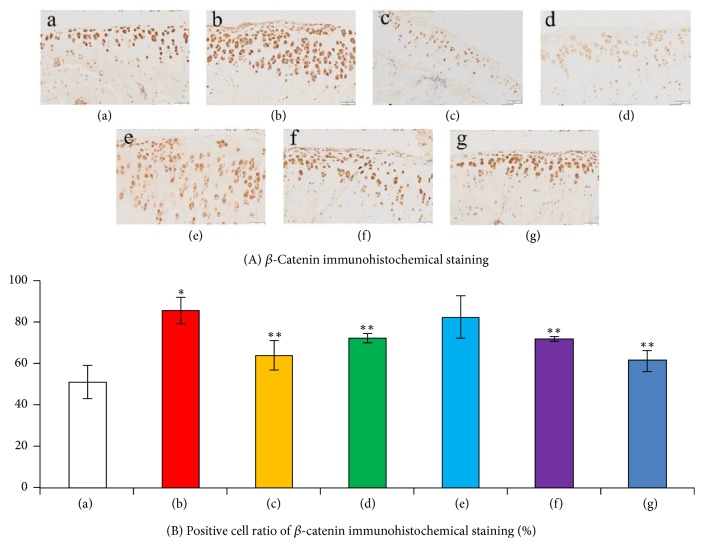
The *β*-catenin immunohistochemical staining results. (A) Immunohistochemistry images of knee joint tissue. (B) Semiquantification of *β*-catenin immunohistochemical staining results. (a) Normal control group; (b) KOA model group; (c) Celebrex group; (d) Viartril-S group; ((e)–(g)) low-dose (e), medium-dose (f), and high-dose (g) QFXBG groups. ^*∗*^*p* < 0.05 versus (a) group; ^*∗∗*^*p* < 0.05 versus (b) group.

To test the activity of down-stream signaling pathways of C/ebp*α* expression, the *β*-catenin immunohistochemical staining was performed. From the results (Figure 7), compared to the normal control group, the expression of *β*-catenin in the KOA model group was significantly upregulated. While compared to the KOA model group, the expressions of *β*-catenin in the medium-dose and high-dose QFXBG groups were significantly decreased.

## 4. Discussion

QFXBGs have shown significant therapeutic efficacy in the treatment for KOA in the past two decades of clinical application in Huadong Hospital Affiliated to Fudan University and Longhua Hospital Affiliated to Shanghai University of Traditional Chinese Medicine. QFXBGs contain multiple components, including* Astragalus mongholicus* Bunge that modulates the immune system, preventing pathogen invasion; tetrandrine methiodide that prevents rheumatism;* Cyathula capitata* Moq that promotes downflow of blood, ground beetle that ameliorates blood stasis and is beneficial for the nervous system; Rhizoma Atractylodis that facilitates excretion of excess water out of the body. Radix Angelica Sinensis which is often used to enrich blood and promote blood circulation; and Herba Epimedii which is used to treat liver and kidney yin deficiency. Therefore, with these comprehensive effects, the QFXBGs used here repaired cartilage and cancellous bone trabeculae in the subchondral bone at different degrees.

The major component of Viartril-S is the glucosamine sulfate, which enters the blood system as glucosamine after oral administration. It alleviates KOA via promotion of proteoglycan synthesis by glucosamine. The proteoglycans are important components of cell membrane and cell interstitial, and they function to stabilize cell membrane and enhance intercellular junction, thereby inhibiting vascular exudation and cell dissociation while promoting the synthesis of collagen in articular cartilage matrix [10]. Supplementation of glucosamine has important significance of cartilage repair especially for patients with KOA, a condition in which the metabolism of articular cartilage is increased, together with increased synthesis and metabolism of collagen, glycosaminoglycans, and other structural proteins. In this study, Viartril-S exerted reparation effect on cartilage compared with the KOA model group, but the effect of glucosamine on subchondral bone remains unknown.

The Celebrex has a major component of celecoxib, which is a selective COX-2 inhibitor. The celecoxib inhibits synthesis of inflammatory factors and decreases protein levels of COX-2 and mPGES-1. The decreased PGE2 synthesis further results in reduced differentiation into chondrocytes and inhibition of bone hyperplasia [11]. The Celebrex is well-absorbed and shows rapid efficacy and serves as an effective analgesic for early-onset and acute KOA. However, for the multifactorial KOA, the mere inhibition of inflammatory factors by Celebrex is insufficient to alleviate cartilage degeneration.

Yiqi-Huayu-Lishui Fang (YQHYLSD) is the original decoction of QFXBG, which is developed as a new commercialized Chinese medicine granule. It is convenient to holding, transportation, and application. The composition of QFXBG and YQHYLSD has the same varieties of herbs. There is only a difference in the dosage forms between them; QFXBG is the granule while YQHYLSD is the decoction. Our previous study had shown that YQHYLSD can significantly inhibit focal PGE2, 6-Keto-PGF1*α*, and TXB2 in tissues [12] and alleviate pain and inflammation, thereby mitigating inflammatory damage to synovial membrane and cartilage. They also inhibit TGF-*β*1 expression in cartilage of KOA and thereby the bone hyperplasia [13]. As an addition to these findings, the results in this study from Micro-CT, HE staining, ABH/OG image, immunohistochemistry, and TUNNEL staining showed that, compared to the KOA model group, the three doses of QFXBGs ameliorated to some degree the degeneration of knee articular cartilage and cancellous bone in the subchondral bone, inhibited generation of inflammatory factors, and decreased chondrocyte apoptosis. In particular, the high dose performed better than the other two and performed comparably to Viartril-S in cartilage repair and slowing down the cartilage degeneration, but superior to it in attenuating degeneration of subchondral bone. The overall efficacy of low and medium doses of QFXBGs was inferior to Viartril-S. When compared to the celecoxib, the high-dose QFXBGs were slightly more effective in attenuating the degeneration of cartilage and subchondral bone, while the medium and low doses performed comparably to celecoxib on the whole.

The articular cartilage is composed of chondrocytes and extracellular matrix. The cartilage lacks vascular system and thus has a quite weak ability to regenerate and repair after injury [14]. There are two types of cartilage injury, partial-thickness cartilage defects and full-thickness cartilage defects. The partial-thickness cartilage defects involve a rather slow repair process, including extracellular matrix increase and fibrosis healing, together with proliferation of chondrocytes around the injury site. In the full-thickness cartilage defects, the defects continue down to the subchondral bone, inducing rupture of small blood vessels located within the medullary cavity of cancellous bone of subchondral bone, resulting in blood clotting on the lesion surface. In the absence of overwhelming loading, the undifferentiated mesenchymal cells migrate into the clot, whereby they keep proliferating and differentiating into chondrocyte-featuring cells to self-repair. However, by this self-repair, fibrous cartilage is formed, which is different from natural cartilage in biochemical formation, structure and mechanical characteristics, and degeneration occurs with time [15]. Generally, a damage with a diameter <3 mm can repair through generation of a transparent cartilage, while a larger damage area would repair by the fibrous tissue. During KOA, chondrocyte apoptosis is enhanced.

The Wnt/*β*-catenin signaling, Fas and Bax signaling play an important role in KOA [14]. Under normal conditions, the activation of Wnt/*β*-catenin signaling promotes differentiation and maturation of chondrocytes, increases expression and activity of various proteases in the matrix, and accelerates the matrix degradation. On the other hand, the expression of proteoglycans and collagen genes is enhanced in chondrocytes to repair the damaged matrix [16]. The Wnt signaling also participates in regulating differentiation of bone marrow mesenchymal stem cells (BMSCs). The Wnt/*β*-catenin signaling plays an important role in the growth, proliferation, differentiation, migration, apoptosis and maintenance of homeostasis of chondrocytes. The Wnt signaling may exert opposite regulations on chondrocytes with different sites and times of source [17]. The C/ebp*α* signaling can regulate the Wnt signaling, thereby directing the differentiation of BMSCs and affecting articular cartilage generation and apoptosis.

C/ebp*α* is present in multiple cell types, and mainly functions to inhibit cell proliferation and stimulate differentiation [18]. It participates in cell proliferation, differentiation, development, and tumorigenesis [19]. C/ebp*α* can regulate multidirectional differentiation of BMSCs through regulating the canonic Wnt/*β*-catenin pathway [20]. The BMSCs have a potent capability of multidirectional differentiation potential and self-proliferation and are a type of multifunctional adult stem cell, differentiating into multiple cell types such as fat cells, osteoblasts, and chondrocytes [21]. Therefore the BMSCs are extremely important in repair and remodeling of bone and cartilage. The Wnt 10b component in the canonic Wnt/*β*-catenin pathway inhibits BMSC differentiation into fat cells, potentiating the differentiation into osteoblasts. Peroxisome proliferator-activated receptor gamma (Ppar*γ*) is a member of the nuclear hormone receptor gene superfamily and is mainly expressed in fat tissues. Ppar*γ* is an importance factor that induces specific gene expression in fat cells and regulates fat cell differentiation [22]. Studies have shown that C/ebp*α* has a positive feedback on Ppar*γ*, the excessive C/ebp*α*, and the overexpressed Ppar*γ*; the two factors cooperate to promote fat cell generation and induce a switch of the Wnt/*β*-catenin pathway favoring osteoblasts to favoring fat cells [23]. When such adipogenic transcription factors as C/ebp*α* and Ppar*γ* are increased, the expression of osteogenic transcription factors like Dlx5, Osterix, and Runx2 is reduced, inducing a switch of Wnt 10b favoring osteogenesis towards favoring adipogenesis [24]. Studies have shown that a transient overexpression of C/ebp*α* and Ppar*γ* would induce remodeling of the fat issue, while long-term overexpression would result in the occurrence of diabetes and obesity [25], both of which are risk factors of KOA [26, 27]. Wang et al. [28] have reported that C/ebp*α* mediates the differentiation of adipose-derived mesenchymal stem cells into osteoblasts through modulating the levels of bone formation protein (BMP) and transforming growth factor-beta (TGF-*β*). Li et al. [29] have reported that the decreased C/ebp*α* promoter methylation would induce C/ebp*α* overexpression, which inhibits the Wnt/*β*-catenin-mediated BMSC differentiation into osteoblasts, favoring the generation of fat cells. This may explain the tiny and disorganized cancellous bone trabeculae in the subchondral bone observed here.

The canonic Wnt/*β*-catenin signaling pathway also has important significance for chondrocyte differentiation. On the one hand, it promotes chondrocyte development. While on the other hand, it is harmful to the mature chondrocytes, inducing their apoptosis. Although *β*-catenin is essential for cartilage differentiation, its overactivation in mature chondrocytes would cause cartilage degeneration, producing KOA chondrocytes [30]. In this study, the reduced C/ebp*α* promoter methylation in KOA model rats may induce C/ebp*α* overexpression, resulting in activation of the Wnt/*β*-catenin signaling pathway; the overactivation of *β*-catenin induces intracellular accumulation of *β*-catenin, thereby promoting cartilage formation and apoptosis of mature chondrocytes. In the presence of KOA, apoptosis exceeds generation for chondrocytes.

In this study, QFXBGs increased methylation of C/ebp*α*-2 CpG island at 10 bp from promoter, and that would be likely to regulate C/ebp*α* expression and promoted cartilage formation while decreasing chondrocyte apoptosis, thereby restoring the cartilage and subchondral bone. Our findings provide insights into the therapeutic treatment of KOA. However, the underlying mechanisms are unclear. It is possible that QFXBGs modulate the Wnt/*β*-catenin pathway through regulating C/ebp*α* expression, thereby regulating the osteogenesis and cartilage-forming effects of BMSCs.

## 5. Conclusion

First, the decreased C/ebp*α*-2 promoter methylation in the CpG island at 10 bp from the start codon may be involved in the pathogenesis and progress of KOA. Second, the QFXBG did have shown ameliorating effects on degeneration of articular cartilage and the subchondral bone, and the high dose performs better than medium and low doses. These effects may be mediated through the elevated C/ebp*α*-2 promoter methylation level, although the elevation is not associated with the QFXBG dose. In sum, this study showed that QFXBG is effective in ameliorating, preventing, and treatment of KOA through promoting C/ebp*α* methylation at specific promoter sites. However, the underlying mechanisms need further studies.

## Figures and Tables

**Table 1:** Primers of target genes.

Primer name	Sequence
Cebpa_2F	TGGAAAGTTATAAGAGAAGGTAGGTTT
Cebpa_2R	CCACCCAATACCCCAACTC
Cebpa_22F	GGATAGAAGGGGTTTGGTGAG
Cebpa_22R	AACCATTACACTAAAAAATCTTAACCTTAC

**Table 2:** Semiquantification of immunohistochemical and TUNNEL staining results.

Groups	Positive cell ratio $\left(\overset{-}{x}\pm s\right)$%						
	Control group	KOA model group	Celebrex group	Viartril-S group	Low-dose QFXBG	Medium-dose QFXBG	High-dose QFXBG
BAX	75.21 ± 8.03	93.55 ± 2.70^a1^	78.30 ± 5.79^b1^	83.27 ± 5.53^c1^	86.46 ± 2.78^d1^	81.31 ± 1.00^e1^	76.30 ± 3.02^f1^
BCL-2	71.45 ± 3.81	87.96 ± 2.62^a2^	77.58 ± 4.27^b2^	77.17 ± 4.94^c2^	86.30 ± 6.71^d2^	86.49 ± 0.92^e2^	80.16 ± 1.28^f2^
FAS	69.24 ± 9.13	89.77 ± 2.69^a3^	80.89 ± 4.52^b3^	77.04 ± 3.76^c3^	79.51 ± 1.49^d3^	75.31 ± 11.83^e3^	80.27 ± 5.21^f3^
TUNNEL	52.94 ± 5.75	79.45 ± 8.04^a4^	73.31 ± 25.23^b4^	68.05 ± 14.16^c4^	78.48 ± 6.81^d4^	54.29 ± 14.09^e4^	61.91 ± 3.40^f4^

*Note*. Compared with control group, ^a1^*p* = 0.0200^*∗*^; ^a2^*p* = 0.0035^*∗*^; ^a3^*p* = 0.0201^*∗*^; ^a4^*p* = 0.0097^*∗*^. Compared with KOA model group, ^b1^*p* = 0.0144^*∗*^, ^b2^*p* = 0.0229^*∗*^, ^b3^*p* = 0.0429^*∗*^, and ^b4^*p* = 0.3349; ^c1^*p* = 0.0443^*∗*^, ^c2^*p* = 0.0289^*∗*^, ^c3^*p* = 0.0088^*∗*^, and ^c4^*p* = 0.2921; ^d1^*p* = 0.0337^*∗*^, ^d2^*p* = 0.7114, ^d3^*p* = 0.0044^*∗*^, and ^d4^*p* = 0.8814; ^e1^*p* = 0.0018^*∗*^, ^e2^*p* = 0.4127, ^e3^*p* = 0.1078, and^e4^*p* = 0.0548; ^f1^*p* = 0.0018^*∗*^, ^f2^*p* = 0.0098^*∗*^, ^f3^*p* = 0.0482^*∗*^, and ^f4^*p* = 0.0253; ^*∗*^*p* < 0.05.

**Table 3:** C/ebp*α* methylation in different groups.

Target gene	Distance from methylation site to the start codon	D versus Y *p* value	Z versus Y *p* value	G versus Y *p* value	Q versus Y *p* value	G versus Z *p* value	G versus D *p* value	Z versus D *p* value
C/ebp*α*-22	1881 bp	0.0088^*∗*^	0.6866	0.0094^*∗*^	0.0115^*∗*^	0.9496	0.1869	0.4858
C/ebp*α*-2	−458 bp	0.3594	0.0005^*∗*^	0.5704	0.0365^*∗*^	0.0042^*∗*^	0.3943	0.9667
C/ebp*α*-2	−452 bp	0.3055	0.1715	0.4011	0.0495^*∗*^	0.2435	0.3781	0.9821

*Note*. ^*∗*^*p* < 0.05.

## Abbreviations

KOA: Knee osteoarthritis
QFXBG: Qi-Fang-Xi-Bi-Granules
HE staining: Haematoxylin & Eosin staining
ABH/OG staining: Orange G staining
EDTA: Ethylene diamine tetraacetic acid
TUNNEL: Terminal-deoxynucleotidyl transferase mediated nick end labeling
C/ebp*α*: CCAAT/enhancer binding protein *α*
Bcl-2: B-cell lymphoma-2
Bax: Bcl-2 associated X protein
Fas: Factor associated suicide
BMSC: Bone marrow mesenchymal stem cell
AMSC: Adipose-derived mesenchymal stem cell.

## Additional Points

*Availability of Data and Materials*. Summarized data have been presented in this manuscript. Raw data for this study are located and protected at Fudan University. Sharing of the raw data is not possible, because a secondary analysis is planned.
